# A Comparison of HWRF, ARW and NMM Models in Hurricane Katrina (2005) Simulation

**DOI:** 10.3390/ijerph8062447

**Published:** 2011-06-23

**Authors:** Venkata B. Dodla, Srinivas Desamsetti, Anjaneyulu Yerramilli

**Affiliations:** Trent Lott Geospatial Visualization Research Center, Jackson State University, 1230 Raymond Road, Jackson, MS 39204, USA; E-Mails: sreeds82@gmail.com (S.D.); anjaneyulu.yerramilli@jsums.edu (A.Y.)

**Keywords:** hurricanes, prediction, numerical models, WRF

## Abstract

The life cycle of Hurricane Katrina (2005) was simulated using three different modeling systems of Weather Research and Forecasting (WRF) mesoscale model. These are, HWRF (Hurricane WRF) designed specifically for hurricane studies and WRF model with two different dynamic cores as the Advanced Research WRF (ARW) model and the Non-hydrostatic Mesoscale Model (NMM). The WRF model was developed and sourced from National Center for Atmospheric Research (NCAR), incorporating the advances in atmospheric simulation system suitable for a broad range of applications. The HWRF modeling system was developed at the National Centers for Environmental Prediction (NCEP) based on the NMM dynamic core and the physical parameterization schemes specially designed for tropics. A case study of Hurricane Katrina was chosen as it is one of the intense hurricanes that caused severe destruction along the Gulf Coast from central Florida to Texas. ARW, NMM and HWRF models were designed to have two-way interactive nested domains with 27 and 9 km resolutions. The three different models used in this study were integrated for three days starting from 0000 UTC of 27 August 2005 to capture the landfall of hurricane Katrina on 29 August. The initial and time varying lateral boundary conditions were taken from NCEP global FNL (final analysis) data available at 1 degree resolution for ARW and NMM models and from NCEP GFS data at 0.5 degree resolution for HWRF model. The results show that the models simulated the intensification of Hurricane Katrina and the landfall on 29 August 2005 agreeing with the observations. Results from these experiments highlight the superior performance of HWRF model over ARW and NMM models in predicting the track and intensification of Hurricane Katrina.

## 1. Introduction

Hurricanes, all over the world, are known to be the most common and most devastating of all the natural disasters. Each year approximately 80–90 tropical cyclones reaching tropical storm intensity occur around the globe, of which about 40–50 attain hurricane intensity of 33 m/s [1]. Data from National Hurricane Center (NHC) show that from 1949 to 1999, 302 hurricanes formed in the Atlantic basin, of which 78 made landfall in the United States and 31 of these hurricanes were intense. The south-east coasts of US are the most vulnerable to the hurricanes of Atlantic Ocean. In association with landfall, coastal regions are the most vulnerable due to strong winds (often exceeding about 100 mph); torrential rains (often exceeding 3 cm/hour and 20 cm/day) and storm surge (often exceeding about 8 ft). Damage due to high wind results in felling trees, building damage, disruption of transport and communication systems; continuous torrential rains for 1–3 days result in flash floods and storm surge lead to coastal inundation all affecting towards loss of life, property and disruption of public life. The estimated cost of losses due to catastrophic hurricanes is about $1.7 billion/ year on average from 1949 to 1988 and $4.2 billion/year from 1989 to 1999, which is attributable to population growth and increasing development in coastal regions. Most of the destruction from the hurricanes occurs during 2–3 days preceding the landfall and few hours after the landfall. All these reasons emphasize the need for improved understanding and prediction of hurricanes, more so the prediction of time and location of landfall and the evolution of hurricanes preceding the landfall.

For hurricane prediction, a variety of predictions models are available as statistical, dynamical, statistical-dynamical, trajectory and ensemble models. Statistical models are based on historical relationships between storm behavior and storm-specific details such as location and date; dynamical models, also known as numerical models, solve the physical equations of motion governing the atmosphere; Statistical-dynamical models blend both dynamical and statistical techniques by making a forecast based on established historical relationships between storm behavior and atmospheric variables provided by dynamical models; Trajectory models move a tropical cyclone (TC) based on the prevailing flow obtained from a dynamical model; and ensemble forecasts are generated by combining the forecasts from a collection of model forecasts.

The track and intensity of hurricanes, although happen simultaneously, are dependent on separate scale processes. While the track is dependent on large-scale processes, intensity changes depend on inner-core dynamics and its relationship with large-scale environment. For these reasons, even coarser grid global models were successful in track prediction [2] whereas high resolution mesoscale models were needed for intensity prediction [3]. Several studies were attempted with grid resolution of a few kilometers (1–10 km) to resolve the characteristic inner core hurricane features like eyewall, eye and rainbands [4–6]. Davis *et al.* [7] studied the intensification prior to landfall of Hurricane Katrina using ARW (Advanced Research Weather Research and Forecasting) Model with 4 km and 1.33 km resolutions and reported that the intensification is sensitive to model resolution and surface momentum exchange and that 1.33 km fine resolution improved the prediction of rapid intensification and the structure of rainbands and that coupling to a mixed layer model reduced the erroneous intensification prior to landfall. In contrast, Shen *et al.* [8] used a mesoscale resolving finite-volume general circulation model with near 25 and 12.5 km resolutions and their results indicated that finer resolution experiment with 12.5 km improved intensity prediction although intensity was overestimated as compared to observations but are comparable to similar WRF simulations with 12 km resolution.

For United States, NHC (National Hurricane Center) provides the official hurricane forecasts using objective guidance provided by different statistical and dynamical models. It is known that dynamical models are most complex based on mathematical formulation of atmospheric dynamics and physical processes and use initial state that can vary tremendously from the real atmosphere leading to uncertainty and forecast errors. Specifically errors in the initial state of a model tend to grow with time during the forecast. As of the current state, numerical models for weather prediction alone can provide the needed quantitative information of meteorological information such as pressure, rainfall and landfall point, which are necessary for the application of decision support systems and initiation of mitigation measures.

In this study, an attempt is made to evaluate the performance of three modeling systems of Weather Research and Forecasting (WRF) model, in the prediction of north Atlantic hurricanes through a case study of hurricane Katrina (2005). WRF model is chosen as it is the latest state of art mesoscale atmospheric model suitable for use in broad spectrum of applications across scales ranging from few meters to 1000’s of kilometers and for the applications in real time prediction and research development. The WRF modeling system, developed as a collaborative effort among the National Centers for Environmental Prediction (NOAA/NCEP), the NOAA Earth Systems Research Laboratory Global Systems Division (NOAA/ESRL/GSD), the National Center for Atmospheric Research (NCAR) Mesoscale Microscale Meteorology Division (MMM), the Department of Defense’s Air Force Weather Agency (AFWA), the Federal Aviation Administration (FAA), and with the participation of a number of university scientists and international collaborators, aims at advancing the understanding and prediction of mesoscale weather. The WRF system offers two separate dynamical cores, the Non-hydrostatic Mesoscale Model (NMM) and the Advanced Research WRF (ARW), and model systems developed for specific applications as HWRF (Hurricane WRF), and WRF/Chem (WRF-Chemistry). In view of the scope of this study for the prediction of Hurricane Katrina, the three modeling systems of ARW, NMM and HWRF were used to make a comparative evaluation of these three model systems in hurricane prediction through a case study of Hurricane Katrina. The authors are aware of nonhomogeneity due to use of different physics, numerics, vortex initialization and lateral boundary conditions (LBCs) for the three models used in the present study. HWRF model uses specific parameterization schemes of physical processes and advanced vortex initialization which are not available with ARW model. Although NMM model has possibility to use some schemes as of HWRF, we used schemes common to ARW and NMM models to facilitate comparison of these two models. This was not possible with HWRF, as some schemes in HWRF are not available in NMM, e.g., GFDL scheme for surface processes. The comparison of these three models is done with this limitation. Perfect LBCs were used with ARW and NMM models as forecast LBCs used in HWRF are not available. The results obtained from present study are to be viewed taking into consideration of these discrepancies.

The case study of hurricane Katrina was taken up as this hurricane was one of the 5 deadliest hurricanes ever to strike US and noted to be one of the most devastating natural disasters in US history. A brief description of hurricane Katrina, models, data and design of experiments is given in Section 2 and the results are presented in Section 3.

## 2. Experimental Section

In this section details of hurricane Katrina, tropical cyclone models, vortex initialization and model design are presented.

### 2.1. Life Cycle of Hurricane Katrina

Katrina was one of the five deadliest hurricanes ever to strike the United States, inflicting catastrophic damage and enormous loss of life in Louisiana and with its effects extending into the Florida, Georgia, and Alabama. Considering the staggering nature of its impacts, Katrina was noted to be one of the most devastating natural disasters in United States history. A detailed description of the life cycle of Hurricane Katrina was provided in a report by Knabb *et al.* [9]. The life cycle of this deadly hurricane occurs during 23–30 August 2005 with landfalls, as Category 1 hurricane on the southeastern coast of Florida at around 2230 UTC 25 August and with Category 3 intensity near the mouth of the Pearl River at the Louisiana/Mississippi border at 1110 UTC 29 August. A description of its life cycle is provided as follows.

**Figure 1.**
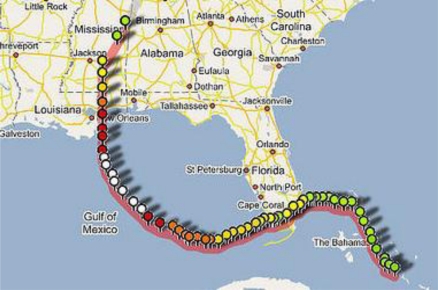
Hurricane Katrina Track—Google Map. (http://www.flickr.com/photos/gisuser/38501841/).

Hurricane Katrina was first identified as a tropical wave on 19 August and as a tropical depression on 23 August situated over southeastern Bahamas. The depression intensified and was designated as the cyclone Katrina at 1200 UTC 24 August with its center located at about 65 nautical miles east–southeast of Nassau. The cyclone system initially moved northwestward and later moved westward on 25 August under the influence of a middle to upper tropospheric ridge over the northern Gulf of Mexico and southern United States. Katrina attained hurricane intensity at around 2100 UTC 25 August, had its landfall with Category 1 intensity on the southeastern coast of Florida around 2230 UTC 25 August and moved west-southwestward over the southeastern Gulf of Mexico. Hurricane Katrina had two periods of rapid intensification between 26 and 28 August, first during 0600 UTC 26 August to 0600 UTC 27 August with maximum winds reaching 95 knots, and the second rapid intensification during 0600 UTC to 1800 UTC 28 August with wind speeds reaching 150 knots making it a Category 5 hurricane. Katrina moved westward on 27 August and northwest on 28 August. Hurricane Katrina had rapid weakening after 1800 UTC 28 August before its landfall near Buras, Louisiana at 1110 UTC 29 August. It continued northward and made its final landfall near the mouth of the Pearl River at the Louisiana/Mississippi border, as a Category 3 hurricane with an estimated wind speed of 105 knots (Figure 1). Katrina rapidly weakened over land to become a Category 1 hurricane by 1800 UTC 29 August, and a tropical storm by 0000 UTC 30 August. The cyclonic system moved northeastward and became a tropical depression at 1200 UTC 30 August. The system, moved northeastward, transformed into an extratropical low pressure system by 0000 UTC 31 August and finally merged within a frontal zone over the eastern Great Lakes.

### 2.2. Tropical Cyclone Prediction Models

In this section, salient features of three modeling systems of the Weather Research and Forecasting (WRF) model suitable for hurricane prediction are presented. All these three models were developed, as multi-agency effort but sourced through NCAR (National Center for Atmospheric Research) and NCEP (National Centers for Environmental Prediction), to provide an advanced mesoscale forecast model system that can be used both as a research tool towards better understanding and prediction of mesoscale weather phenomena and for real time operational forecasting.

Hurricane Weather Research and Forecast System (HWRF) Model: An advanced hurricane prediction system, Weather Research and Forecast system for Hurricane prediction (HWRF), was developed at the NCEP’s Environmental Modeling Center (EMC) for understanding the hurricane forecast issues and improvement of hurricane prediction. Scientific documentation of the HWRF model is provided in a report by Gopalakrishnan *et al.* [10]. This HWRF modeling system, based on the NMM (non-hydrostatic mesoscale model) core of the WRF (Weather Research and Forecasting) model, is a high resolution coupled air-sea-land prediction model with a movable nested grid and with the applicability to the prediction problems of hurricane track, intensity, structure, and rainfall. The model uses a two-way interactive movable nested grid that follows the forecasted path of tropical cyclone. The configuration of HWRF system consists of outermost (parent) domain and the movable nested grid with resolutions of 27 and 9 km respectively and 42 vertical levels. This HWRF modeling system is used for real-time operational forecasts at NCEP since 2007 and provided open access for research community in April, 2010. HWRF uses a non-hydrostatic system of equations on a rotated latitude/longitude Arakawa-E grid, with the parameterization schemes of Simplified Arakawa Schubert (SAS) scheme for cumulus convection, Ferrier (new ETA) scheme for cloud microphysics, GFS non-local planetary boundary layer scheme with surface layer physics and GFDL schemes for longwave and shortwave radiation processes. The operational HWRF model at NCEP is coupled to a high-resolution Princeton University 3-D ocean model (POM) for Atlantic basin hurricane prediction. More details of the formulation and application of HWRF model are provided by Gopalakrishnan *et al.* [11] and Tallapragada *et al.* [12]. The initial and boundary conditions are taken from GFS model global forecasts, which are subjected to relocation and bogussing of the vortex structure [13] using Grid Point Statistical Interpolation (GSI) 3DVAR (three-dimensional variational) data assimilation method described in Section 2.3.

Advanced Research WRF (ARW) Model: The Advanced Research WRF (ARW) modeling system was developed and sourced from National Center for Atmospheric Research (NCAR), as suitable for a broad range of applications. This model system has versatility to choose the domain region of interest; horizontal resolution; interactive nested domains and with various options to choose parameterization schemes for convection, planetary boundary layer (PBL), explicit moisture; radiation and soil processes. ARW is designed to be a flexible, state-of-the-art atmospheric simulation system that is portable and efficient on parallel computing platforms and a detailed description was provided by Skamarock *et al.* [14]. The model consists of fully compressible non-hydrostatic equations and the prognostic variables include the three-dimensional wind, perturbation quantities of pressure, potential temperature, geo-potential, surface pressure, turbulent kinetic energy and scalars (water vapor mixing ratio, cloud water etc). The model equations are formulated using mass-based terrain following coordinate system, and solved in Arakawa-C grid using Runge–Kutta third order time integration techniques. The model has several options for spatial discretization, diffusion, nesting and lateral boundary conditions. ARW supports horizontal nesting that allows resolution to be focused over a region of interest by introducing an additional grid (or grids) into the simulation with the choice of one-way and two-way nesting procedures.

Non-hydrostatic Mesoscale Model (NMM): The Nonhydrostatic Mesoscale Model (NMM) core of the Weather Research and Forecasting (WRF) system was developed by the National Oceanic and Atmospheric Administration (NOAA)/ National Centers for Environmental Prediction (NCEP). A description of the technical aspects of this model is given Janjic *et al.* [15]. The NMM system consists of a dynamics solver, which includes algorithms for computation of pressure gradient and Coriolis force terms and mass divergence, advection schemes, thermodynamic processes, a non-hydrostatic add-on module, horizontal diffusion, and divergence damping. This system has one-way and two-way nesting capability and various options for physics, initialization and post processing produce an end-to-end mesoscale simulation. This model system uses full compressible equations split into hydrostatic and non-hydrostatic contributions, which facilitate easy comparison of hydrostatic and non-hydrostatic solutions with reduced computational effort at lower resolutions. The vertical coordinate uses terrain-following hydrostatic-pressure based sigma coordinate up to a specified pressure surface usually below the tropopause, and hydrostatic pressure coordinate above with Lorenz staggering grid and horizontal coordinate follows rotated latitude-longitude system with Arakawa E-grid staggering. This spatial discretization conserves mass, momentum, energy and enstrophy. The time integration method is forward-backward for fast waves, implicit for vertically propagating sound waves, Adams-Bashforth for horizontal advection and Coriolis terms, Crank-Nicholson for vertical advection, and Lagrangian upstream passive substance advection with forced conservation. The boundary conditions at the top of the model atmosphere uses the vertical velocity to be zero and with hydrostatic and non-hydrostatic pressures to be equal and the bottom boundary conditions as the vertical velocity and the vertical derivative of the difference between the non-hydrostatic and hydrostatic pressures to be zero at the lowest sigma surface. The model has full physics options with parameterizations for the physical processes of land surface, cumulus convection, cloud microphysics, surface physics, planetary boundary layer and free atmospheric turbulence and atmospheric radiation.

### 2.3. Vortex Initialization

All the atmospheric models to be used for the specific purpose of hurricane prediction are to be initialized so that the initial state of the model atmosphere has a realistic cyclone vortex (*i.e.*,) with the properties of the model cyclone system nearer to the observations. Due to lack of observations over oceans where tropical cyclones form and develop, the model initial conditions derived from global/regional analyses based on observations does not truly represent the characteristics of the observed cyclone system. For accurate prediction of the development of these cyclone systems, vortex initialization with right location and realistic intensity is needed. This procedure is often referred to as “bogussing the vortex” in numerical weather prediction for hurricanes. A number of studies have shown that the proper initialization of vortex could significantly improve the intensity and track of tropical cyclones [16,17]. A brief description of the vortex initialization procedure adapted in the three different models is presented in this section.

HWRF model uses a vortex initialization and relocation algorithm based on observed tropical cyclone position and intensity parameters. These parameters are provided operationally in real-time by National Hurricane Center (NHC) for the Atlantic and Eastern Pacific basins. The Grid point Statistical Interpolation (GSI) 3DVAR assimilation was implemented for the vortex initialization as a potential improvement for bogussing the vortex. The relocation procedure allows for vortex relocation and intensity adjustment to better match the actual tropical cyclone observations. The usage of real-time data in the HWRF hurricane core, together with the higher resolution of the model, allows for more accurate hurricane predictions of intensity and structure. A composite synthetic vortex is inserted in the storm location (based on observed estimates) proceeded by the data assimilation. The initialization of tropical cyclones in the HWRF model consists of four major steps: (1) interpolate the global analysis fields from the Global Forecast System (GFS) onto the operational HWRF model grids; (2) remove the GFS vortex from the global analysis; (3) add the axi-symmetric synthetic vortex (constructed based on a series of HWRF model forecasts); and (4) add through data assimilation any available observational data in the vicinity of tropical cyclone area. Steps 3 and 4 provide major advancements over the conventional GFDL tropical cyclone initialization procedure [18].

ARW model has a module for vortex initialization (*i.e.*,) insertion of a bogus vortex which is similar to that used in MM5 model [19]. The methodology has two components, first is detection and removal of cyclone vortex from the analysis field and the second is to blend the newly computed vortex with the background analysis. The first step is necessary as the vortex contained in the coarse resolution analyses is too broad and too weak and with erroneous center location. The second part of adding the bogus storm to the background field is achieved by designing an axi-symmetric vortex and then adding it to the background analysis. The designed bogus storm is axi-symmetric and will not affect the storm motion. The input data to the bogussing scheme consists mainly of storm location and estimated maximum winds. The bogus storm profile chosen here is based on the following assumptions: (1) Axi-symmetry; (2) Vorticity specified within 400 km of the bogus storm center; (3) Radius of maximum wind (RMW) fixed (120 on 27 km grid); (4) Mass and wind fields in non-linear balance; (5) Nearly saturated core (with respect to water or ice); no eye (on 27 km grid); and (6) Maximum winds of bogus storm are a pre-determined fraction of maximum winds observed. The specification of a three-dimensional vortex structure is arbitrary.

NMM model does not have a module for vortex initialization as part of model code. For this reason, we have used the “REGRIDDER” module with bogus vortex option available with MM5 model to create the intermediate files which contain the 3-D meteorological and surface pressure fields. These are used along with the intermediate files created from “UNGRIB” program to run “METGRID” program of NMM-WPS module to obtain the initial fields which has bogus vortex. The specification of parameters necessary for creating a bogus vortex in MM5 model is same as that of ARW model. However differences in the initial conditions may arise due to blending and interpolation of the intermediate files from “MM5-REGRIDDER” with “NMM-UNGRIB” files.

For both ARW and NMM models, maximum winds of the bogus storm are a user specified fraction (α) of the observed maximum winds. The wind profile of the vortex is given by a Rankine vortex. The altitude factor for this set up is 1.0 from surface to 850 hPa; 0.95 from 850 to 700 hPa; 0.9 from 700 to 500 hPa; 0.7 from 500 to 300 hPa; 0.6 from 300 to 200 hPa and 0.1 from 200 hPa to top. The relative humidity is defined as nearly saturated within the radius of maximum winds; not affected outside twice the radius of maximum winds; and linearly weighted in between.

### 2.4. Model Design and Numerical Experiments

**Figure 2.**
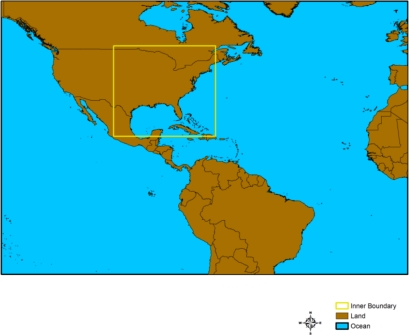
Model domains—outer domain (27 km) and inner domain (9 km).

All the three models, HWRF version 2.0, ARW version 3.2 and NMM version 3.2 were adapted to the study area covering North Atlantic Ocean and adjoining land regions of North and South American continents. The models are designed to have two-way interactive nested domains with 27 and 9 km resolutions as shown in Figure 2. ARW and NMM models have the 9-km inner domain as fixed, whereas HWRF has the 9-km domain to move with the center of the storm during the model integration period (section 3). The initial and the time varying boundary conditions are interpolated from NCEP FNL for ARW and NMM models and from NCEP GFS forecast fields for HWRF model. HWRF model outputs were provided by EMC/NCEP, which were produced using NCEP GFS data. Since these GFS data for the study dates are not available, FNL global analysis has been used. NCEP FNL (Final) Operational Global Analysis data (http://dss.ucar.edu/datasets/ds083.2/) are provided operationally at 6-hour interval at 1.0 degree resolution whereas NCEP GFS (http://www.nco.ncep.noaa.gov/pmb/products/gfs/) is a global spectral data assimilation and forecast model system providing data at 6-hour time interval at a resolution of 0.5 degree (GFS native resolution T254). Both the FNL and GFS uses the same model with the FNL prepared about an hour after the GFS is initialized with assimilation of more observational data. GFS is run earlier to meet forecast needs, and uses the FNL from the previous 6-hour cycle as part of its initialization. Both FNL and GFS are provided at the surface, at 26 mandatory pressure levels from 1,000 hPa to 10 hPa, and at other significant levels. For the present study of Hurricane Katrina, bogus vortex module has been activated with the values of location as 24.6N, 83.3W and maximum wind as 46 m/s which are same as NHC reports and the chosen values radius of maximum wind (RMW) as 120 km and α (Vmax ratio) as 0.75. The choice of RMW was based on QuickSCAT and FNL 10 m winds and 0.75 for α is based on several simulations of hurricanes with different intensities and grid increments [19].

Following the life cycle of the Hurricane Katrina (Section 2) and due to its final landfall around 1110 UTC on 29 August, model integrations were carried out for 72-hours starting from 0000 UTC of 27 August 2005, with the main aim of predicting the intensity changes and track within the three days prior to the landfall. The model design and the choice of parameterization schemes as used for the present study are given in Table 1.

## 3. Results and Discussion

The results from the model integrations with ARW, NMM and HWRF models are presented in this section. As mentioned in Section 2.3, vortex initialization was done with the appropriate schemes leading to some differences in the initial conditions for the respective integrations. The salient differences in the characteristics of the model vortex at 0000 UTC of 27 August 2005 are first described, followed by the results of model integration in terms of the hurricane intensification and movement.

**Figure 3.**
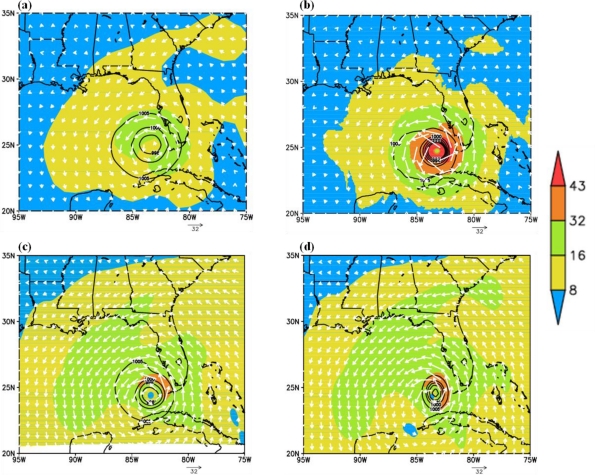
Horizontal distributions of 925 hPa wind speed (m/s) (shaded); sea level pressure (hPa) (contour), and wind vector as white barb associated with the initial vortex at the model initial time of 0000 27 August 2005 with **(a)** FNL analysis, **(b)** HWRF model, **(c)** ARW model, **(d)** NMM model.

Characteristics of the initial vortex: At 0000 UTC of 27 August 2005, corresponding to the initial time of model integration, the sea level pressure (hPa) distribution and wind strength (m/s) and flow fields at 925 hPa for the HWRF, ARW and NMM models and NCEP FNL analysis are presented in Figure 3. In this figure, isobars are shown as contours drawn at 5 hPa interval and with the outermost isobar as 1005 hPa; wind strength is shown as shaded with intensities of 8, 16, 32 and 43 m/s which correspond to threshold values of depression, tropical cyclone, Category-1 and Category-2 hurricanes and wind flow is shown as velocity barbs with the arrowhead pointing the direction of flow. The sea level pressure distribution shows concentric isobars with minimum central surface pressure values of 964 hPa for HWRF; 994 hPa for ARW and NMM; and 996 hPa in FNL analysis. The structure of isobars, as noted from the pattern of 1,005 hPa isobar, shows elongated pattern in all the model fields whereas it is nearly circular with about 250 km radius in FNL analysis. ARW and HWRF models show elongation towards west and NMM model shows elongated isobars to north and south. The corresponding west/east distances are 350/200 km for ARW model; 350/100 km for HWRF and north-south and east-west distances are 100 and 150 km respectively for NMM model, indicating the differences in asymmetries in the vortex at the initial time. Of all the three, NMM model has the smallest vortex in horizontal size, and HWRF has the largest pressure gradient due to the minimum central sea level pressure value of 965 hPa. As of 925 hPa level winds, both ARW and NMM models show maximum winds of 39 m/s and 40 m/s (Cat-1 hurricane winds) with the RMW (radius of maximum wind) as 174 km and 81 km respectively. In contrast, HWRF has maximum wind of 43 m/s (Cat-2 hurricane winds) at 70 km radius (RMW) and FNL analysis show maximum wind of 25 m/s (tropical storm winds) at 188 km radius (RMW). ARW and NMM models show concentric flow of tropical storm winds (16–32 m/s) more towards northwest with maxima (Cat-1 strength) towards north, whereas HWRF has nearly circular concentration of tropical storm winds (16–32 m/s) with Cat-2 maximum strength all around the center. FNL has maximum strength of tropical storm winds and mostly circular in shape. In comparison, HWRF model has the maximum wind of 43 m/s closer to the observed value of 46 m/s as compared to 37 m/s with ARW and 40 m/s with NMM model.

**Figure 4.**
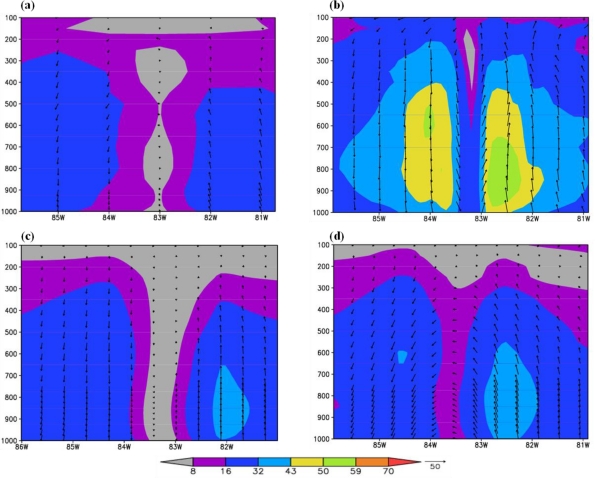
Longitude- vertical sections of horizontal wind with magnitude (shaded) and wind vectors (black barbs) at the model initial time of 0000 UTC 27 August 2005 **(a)** along 25N-FNL analysis, **(b)** along 24.7N-HWRF model, **(c)** along 24.3486N-ARW model, **(d)** along 24.785N-NMM model.

The longitude-vertical cross section of the horizontal wind along the latitude of the vortex center for FNL and the three models are presented in Figure 4. It is noted that the vortex center location for the three models are 24.3486N, 83.4324W for ARW; 24.785N, 83.325W for NMM and 24.7N, 83.2W for HWRF as compared to the observed location at 24.6N, 83.3W and the corresponding track positional errors are 16, 21 and 32 km for HWRF, NMM and ARW models respectively. However the wind structure shows significant differences. The vortex in FNL analysis has a smaller core with larger radius of maximum wind (8–16 m/s) with radial extent up to 1 degree and vertically extending up to 400 hPa and outflow above. HWRF has core wind strength of 43–50 m/s extending up to 400 hPa level either side and with maximum winds of 50–59 m/s extending up to 650 hPa on east side and between 650–520 hPa levels towards west; ARW model has inner core winds of <8 m/s and with maximum winds (32–34 m/s) on the east side and height of maximum extending up to 650 hPa level; and NMM has inner core winds of 8–16 m/s with maximum winds of 32–43 m/s towards the east and with the maximum winds extending up to 550 hPa level. The significant differences of the structure of vortex are higher core and maximum strength winds in HWRF model and smaller inner core with higher maximum winds with higher vertical extent than ARW model.

**Figure 5.**
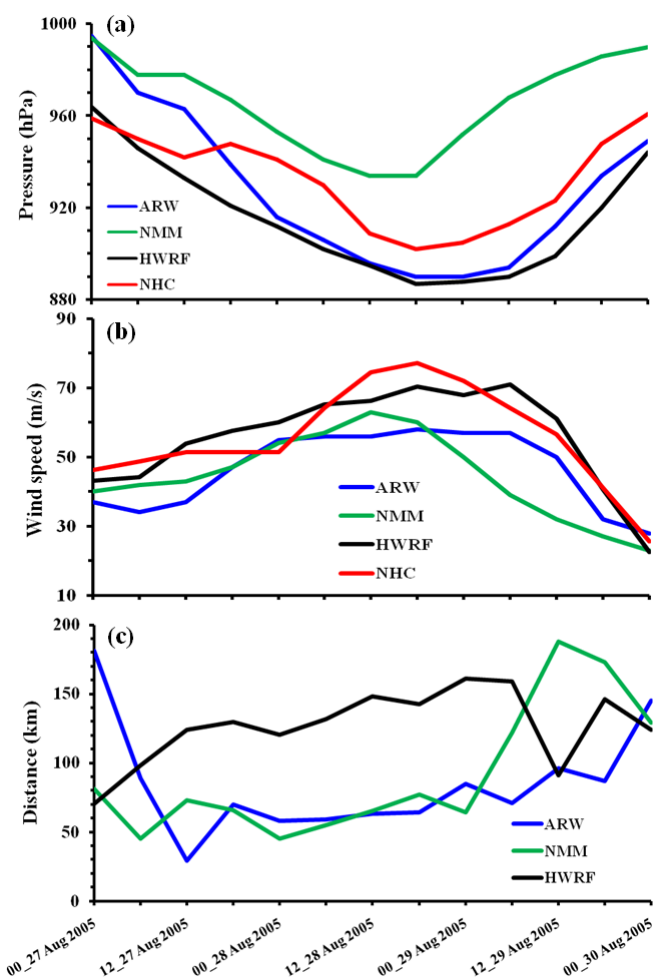
Time series of model predicted **(a)** central sea level pressure (hPa) **(b)** maximum wind (m/s) and **(c)** radius of maximum wind (km) for the experiments with ARW, NMM and HWRF models. NHC estimates of CSLP and MW are noted along with model predicted time series.

Prediction of intensity: The simulation of the evolution of Hurricane Katrina during the model integration period (*i.e.*,) from 0000 UTC of 27 August to 0000 UTC of 30 August, 2005 with the three models are presented (Figure 5) in terms of the time series of central sea level pressure (CSLP), 10-m level maximum wind (MW) and the radius of maximum wind (RMW). The time series of the simulated CSLP along with observations are shown in Figure 5a. At the initial time, the CSLP in HWRF is nearly 965 hPa as of the observations whereas it is 995 hPa for the NMM and ARW models. This is one of the discrepancies in the characteristics of the initial vortex as initialized in the three models. All the three models simulate rapid deepening with the attainment of minimum CSLP around 36–42 hours. Although ARW model has higher CSLP at the initial time as compared to HWRF, both HWRF and ARW attain the same minimum value for CSLP as 890 hPa at 42 hour (*i.e.*,) 1800 UTC of 28 August. Both HWRF and ARW simulate stronger hurricane than of the observations (940 hPa). But the time of attainment of minimum with ARW and HWRF models agrees with the observations. In comparison, NMM simulates a weaker hurricane with attainment of minimum CSLP of 940 hPa at 36 hour (*i.e.*,) 1200 UTC of 28 August, 2005. All the three models simulate gradual weakening of hurricane after 1800 UTC of 28 August, 2005 as similar to the observations. It is significant to note from the life cycle of hurricane Katrina that the minimum CSLP slightly increased between 1200 UTC and 1800 UTC of 27 August, 2005 and correspondingly ARW and NMM models simulated reduced intensification retarded 6 hours (0600 to 1200 UTC) whereas HWRF did not show this feature. However, NMM model shows maintenance of same intensity and ARW a reduction in the rate of deepening during a 6 hour period between 0600–1200 UTC of 27 August, 2005 (*i.e.*,) weakening period 6-hours earlier than observation.

The model simulated maximum wind at 10 m level from the three models along with NHC estimates are shown in Figure 5b. The time variation of MW show that all the three models have the initial strength of the vortex to be slightly lower (37 m/s for ARW, 40 m/s for NMM and 43 m/s for HWRF) as compared to the NHC designated MW of 46 m/s.

These features of MW, minimum CSLP and the horizontal scale of hurricane vortex show that both HWRF and ARW models have nearly same horizontal size with stronger pressure gradients in HWRF as compared to NMM model with smaller horizontal size and pressure gradients higher than ARW model and less than HWRF model. NHC designated MW show slow intensification for 24 hours up to 0000 UTC of 28 August, rapid increase between 24–42 hours attaining a maximum of 75 m/s followed by rapid decrease. ARW predicts intensification from 12 to 24 hours, attaining a maximum of 58 m/s at 42 hours (1800 UTC 28 August), and decreases gradually after 54 hours. NMM model shows gradual increase up to 12 hours, then a steady increase up to 36 hours attaining a maximum of 63 m/s, followed by rapid decrease. In contrast, HWRF shows same intensity for first 6 hours, followed by gradual increase up to 42 hours attaining a maximum of 70 m/s maintained same intensity between 42–54 hours, followed by rapid decrease.

Of the three simulations, HWRF model is better with respect to the rapid intensification although time of attainment of MW is delayed by 12 hours. NMM shows better rate of intensification than HWRF but with underestimation of the maximum intensity. In contrast, ARW model simulated minimum CSLP as of HWRF but with lesser MW indicating a larger hurricane system with weaker pressure gradients.

The simulated RMW from the three models are shown in Figure 5c. HWRF and NMM have the same RMW with a value of 70–80 km at the initial time as compared to 170 km in ARW model. HWRF simulates an increase of RMW up to 12 hours, and a slow increase between 12–48 hours reaching 160 km at 48 hours, remains same between 48 and 54 hours, followed by a decrease. In contrast, ARW model simulates rapid decrease during first 12 hours attaining 30 km as RMW, increase between 12 and 18 hours reaching 70 km, nearly same RMW of 70 km between 18–42 hours, and a gradual increase thereafter. NMM model simulated a decrease during first 6 hours reaching a value of 45 km, an increase in next 6 hours reaching 70 km followed by a decrease up to 24 hours reaching 50 km, a gradual increase up to 48 hours reaching 70 km, rapidly increasing to 180 km at 60 hours and a decrease thereafter. The time variations of RMW are typically different in the three simulations. NMM model maintain nearly the same RMW with minor fluctuations up to 48 hours followed by a rapid increase indicating slower changes in intensity up to 48 hours in association with gradual intensification. HWRF shows gradual increase of RMW up to 54 hours indicating increase of horizontal size in association with a mature stage hurricane. In contrast, ARW model simulated intensification during the first 12 hours as indicated by the shrinking of RMW and increase of horizontal size between 12–48 hours in association with the expansion of the hurricane system.

The typical values of minimum CSLP and MW and RMW indicated the differences in the characteristics of the initial vortex and their impact on the evolution of hurricane Katrina.

**Figure 6.**
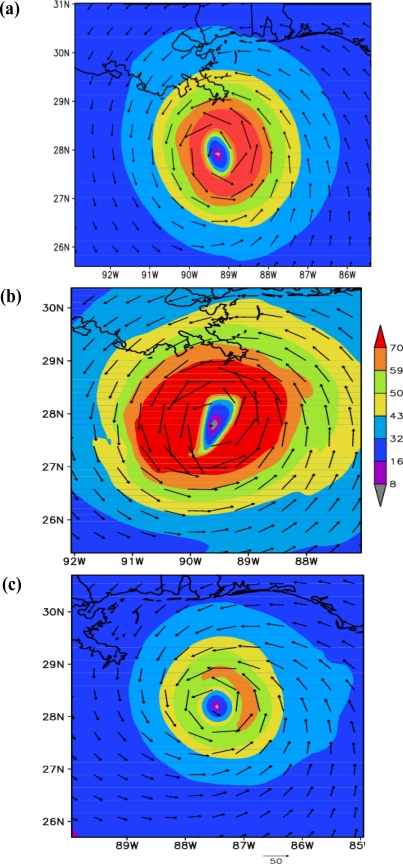
Horizontal wind flow (black vectors) and wind magnitude (m/s-shaded) at 925 hPa level corresponding to the time of mature stage for **(a)** HWRF at 1200 UTC 28 August 2005, **(b)** ARW at 2100 UTC 28 August 2005, **(c)** NMM at 1200 UTC 28 August 2005.

**Figure 7.**
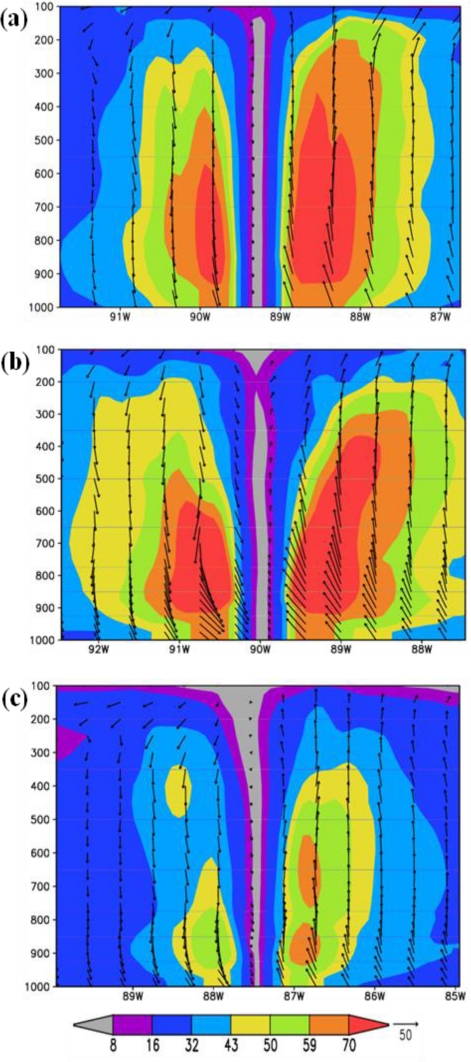
Longitude- vertical sections of horizontal wind with magnitude (m/s-shaded) and wind vectors (black barbs) at the mature of stage of model hurricane for (a) HWRF at 1200 UTC 28 August 2005 along 27.86N **(b)** ARW at 2100 UTC 28 August 2005 along 27.8757N **(c)** NMM at 1200 UTC 28 August 2005 along 28.2N.

Structure of the vortex at mature stage: The characteristics of the hurricane at the mature stage for the experiments with ARW, NMM and HWRF models are shown in Figures 6 and 7. The times of mature stage are based on the time of attainment of maximum intensity in terms of minimum central sea level pressure and maximum surface wind, which are 1200 UTC 28 August 2005 for HWRF and NMM models, and 2100 UTC 28 August 2005 for ARW model. The plots in Figures 6 show horizontal wind flow with wind magnitude at 925 hPa level corresponding to the respective times of mature stage. HWRF model (Figure 6a) simulated maximum winds exceeding 70 m/s at a radius of 50 km and winds are more symmetric than ARW and NMM model simulated winds. ARW model (Figure 6b) simulated winds exceeding 70 m/s with a noted maximum value of 82 m/s at radius of 117 km and the wind asymmetry show larger horizontal extent towards east. NMM model simulated (Figure 6c) maximum winds of 63 m/s at a radius of 65 km and the horizontal structure is more symmetrical than ARW with asymmetries as slightly larger horizontal extent towards east. The plots in Figure 7 show the longitude-vertical sections of wind vectors (barbs and magnitude) along the latitudes of 27.86N for HWRF, 27.8757N for ARW and 28.2N for NMM models. HWRF model simulates narrow eye region through the vertical extent up to 150 hPa and with hurricane winds >70 m/s over a larger area towards east extending up to 400 hPa on the east side and 600 hPa towards west (Figure 7a). ARW model simulated a narrow eye region with surrounding eye wall and with winds exceeding 70 m/s extending up to 350 hPa level on the eastside and 550 hPa on westside (Figure 7b). The horizontal and vertical extent of maximum winds >70 m/s are slightly more in ARW model as compared to HWRF model. NMM model also simulated a narrow eye region surrounded by core of stronger winds with wind maximum of 59–70 m/s towards east and 50–59 m/s towards west (Figure 7c). The vertical extent of 50–59 m/s winds extended up to 450 hPa on the eastern side as compared to 750 hPa on the western side. These features indicate that HWRF and ARW models simulated a hurricane with nearly same horizontal and vertical extent and larger than NMM model.

**Figure 8.**
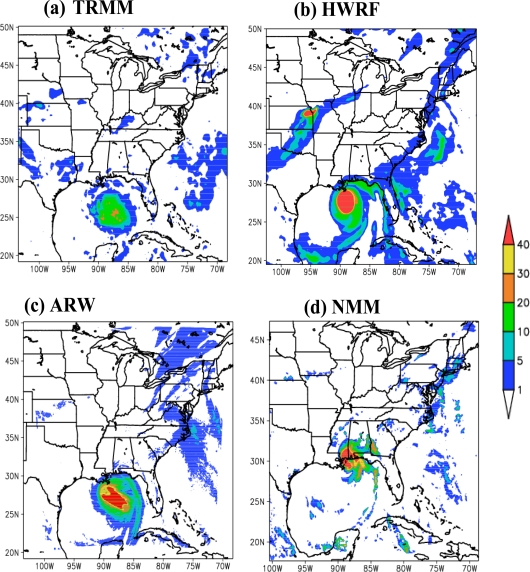
Model predicted 24-hour accumulated rainfall (cm) during the period from 0000 UTC of 28 to 0000 UTC of 29 August 2005 along with corresponding observed rainfall (cm) from TRMM.

Predicted rainfall distribution: The spatial distribution of model simulated rainfall during the 24 hour period between 0000 UTC of 28 and 0000 UTC of 29 August, 2005 for the ARW, NMM and HWRF models along with TRMM observations are shown in Figure 8. ARW model simulated concentrated regions of rainfall, with magnitude exceeding 40 cm, covering north Atlantic and adjoining coastal regions of Texas, Louisiana, Mississippi and Florida. The NMM model predicted concentrated rainfall exceeding 40 cm over coastal regions of Louisiana and Mississippi; and HWRF predicted all the rainfall over the North Atlantic Ocean with the maximum exceeding 30 cm to the west of Florida coast. As of the spatial distribution, HWRF produced the maximum towards east; ARW model to the west and NMM model to the north with respect to TRMM observations. ARW and NMM models overestimated the intensity whereas HWRF simulated smaller horizontal extent. The differences in the location of maximum of the three models are due to differences in the time and location of landfall. It is to be noted that HWRF has the landfall around 1000 UTC of 29 August near the location (29.4N, 89.8W) as of the observations, NMM has the landfall around 0000 UTC of 29 August at the location (30.43N, 88.35W) which is to the east of the observations and ARW model shown the landfall at 0900 UTC of 29 August and at the location (29.35N, 91.04W), which is to the west of the observed landfall.

**Figure 9.**
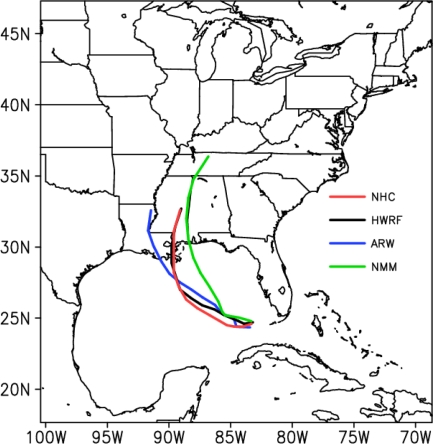
Predicted track positions of Hurricane Katrina from ARW, NMM and HWRF models along with observed track positions from US National Hurricane Center.

Prediction of the track: The model simulated track of hurricane Katrina with the three models along with the best track positions from NHC are shown in Figure 9. Due to vortex initialization, the positions of initial vortex are nearly same in all the three models. However, there are differences in the structure which are described earlier in this section. Of all the three experiments, HWRF produced the best track with the track positions nearly same during the 48 hour period before the landfall and slightly towards east of the observed during the initial 24-hours. ARW model shows deviation of the track towards right of the observations during initial 24-hour period and gradually moving towards west of the observations after 36 hours with the final landfall over Texas coast that is towards west of the observed landfall. In contrast, NMM model shows deviation of the track towards east all through with the landfall over Mississippi coast (*i.e.*,) to the east of the observations.

**Figure 10.**
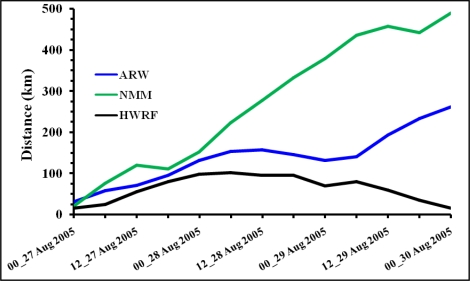
Vector Track Position Errors (km) for the model experiments with ARW, NMM and HWRF models.

The vector track position errors at different times with different models are shown in Figure 10 and values are given in Table 2. HWRF has the minimum errors with values not exceeding 102 km and consistently less than 90 km during the last 24 hours of integration indicating the speed and track of hurricane Katrina closer to the observations. The errors with ARW model gradually increase reaching 132 km at 24 hours and 262 km at 72 hours whereas NMM model has the largest errors of 150 km at 24 hour and 490 km at 72 hours. These error statistics indicate that the NMM model has a higher speed of movement than ARW model contributing to larger track position errors.

## 4. Conclusions

The life cycle of hurricane Katrina (2005), during the three days prior to its landfall was studied using ARW, NMM and HWRF modeling systems of the Weather Research and Forecasting mesoscale model. Since the three models used different procedures for vortex initialization and different schemes for physics and numerics, the differences in the characteristics of the vortex at the initial time and intensification and movement during the 3 day period from 0000 UTC of 27 August to 0000 UTC of 30 August, 2005 were analyzed. The model predictions were validated by comparison with NHC reports. The results obtained are summarized as follows.

- The results indicate that vortex initialization is necessary for hurricane prediction. NCEP FNL global operational analysis shows a weaker hurricane as compared to NHC reports. All the three models show better features of the initial vortex than FNL due to the adapted vortex initialization schemes.
- HWRF model produced the features of initial vortex as best among the three simulations. HWRF simulated minimum central sea level pressure of 964 hPa and 10 m maximum wind of 43 m/s as compared to 994 hPa and 37m/s with ARW and 994 hPa and 40 m/s with NMM models.
- HWRF has largest pressure gradients; NMM has the smallest horizontal extent; HWRF has larger area of hurricane scale (>32 m/s) winds. ARW has same vortex size as HWRF but with lesser intensity.
- HWRF produced stronger core winds with higher vertical extent as compared to ARW and NMM models. NMM had slightly stronger core and higher vertical extent than ARW.
- HWRF and ARW models simulated lower central surface pressure (890 hPa) than observed as compared to higher central surface pressure (940 hPa) with NMM.
- As of 10 m maximum wind at mature stage, HWRF simulated the highest magnitude of 71 m/s which is closer to the observed value of 77 m/s as compared to 63 m/s with NMM and 58 m/s with ARW models. HWRF simulates larger radius of maximum wind as ∼160 km, about twice the RMW of 70 km with ARW and NMM models.
- At the mature stage, both HWRF and ARW models simulated nearly same vertical extent of the Cat-4 and Cat-5 hurricane scale winds reaching up to 300 hPa and with vertical extent higher towards east of the center. In comparison, NMM model had Cat-4 winds limited to the layer between 900–500 hPa.
- HWRF simulated heavy rainfall in the hurricane core region with clear depiction of rain band formation. ARW model simulated heaviest rainfall in the core region but oriented as northwest to southeast. NMM had a smaller region of heavy rainfall as compared to HWRF and ARW models. The locations of the heavy rainfall region differ due to the differences in the landfall point. As such, HWRF simulated the location of the rainfall nearer to the observations.
- HWRF produced the best track nearly coinciding with the NHC reported observations. NMM shows deviation of track towards right and ARW model shown deviation towards left of the observations. HWRF has the least of the track errors not exceeding 100 km in 72 hours as compared to increasing errors reaching 200 km and 400 km with ARW and NMM models respectively.

The authors are aware that this is a single case study restricted to hurricane Katrina and the derived results may vary for other hurricanes. The above described simulated features of hurricane Katrina with HWRF, ARW and NMM models show that HWRF model has the best vortex initialization and produces the best intensification and movement. ARW model produces slightly better simulation than NMM both in terms of initial vortex and intensification and movement. These results show the advantages of a specialized model for hurricane studies incorporating better scheme for vortex initialization and use of moving the inner nest synchronous with the center the model hurricane. Although this is a single case study, the advanced vortex initialization in HWRF model shows merit over the vortex initialization adapted in ARW and NMM models. This is evident with the central sea level pressure, maximum wind and radius of maximum wind in HRWF to be closer to observations, whereas the CSLP do not match in ARW and NMM models. The authors suggest that HWRF simulation is better compared to the other two because of better vortex initialization and better model performance which may be due to coupling with an ocean model.

Although NMM and HWRF model use nearly same dynamics, better simulation of the hurricane structure and intensification and track positions are attributable to ocean-atmosphere interaction and moving nest option to a lesser extent. ARW model has shown some advantages in terms of simulating stronger intensity than HWRF although with a slight overestimation than observation. Numerical experiments with ARW model with moving nest option and with higher horizontal resolution are being attempted as further study.

## Figures and Tables

**Table 1.** Design of the model experiment.

**Model**	ARW/NMM		HWRF	
**Dynamics**	Primitive equation, non-hydrostatic		Primitive equation, non-hydrostatic	
**Vertical resolution**	42 levels		42 levels	
**Domains**	Domain 1	Domain 2	Domain 1	Domain 2
**Horizontal resolution**	27 km	9 km	27 km	9 km
**Domains of integration**	141.9 W–2.88647 W 28.1 S–65.427 N	103.5 W–68.63108 W 19.2 N–50.1324 N	141.9 W–2.88647 W 28.1 S–65.427 N	Moving
**Radiation**	Dudhia scheme for shortwave RRTM scheme for long wave		GFDL for short wave and long wave Radiation	
**Cumulus convection**	Kain-Fritsch (new Eta)		Simplified Arakawa- Schubert	
**Explicit moisture**	WSM6		Ferrier	
**PBL**	Mellor-Yamada-Janjic TKE		NCEP GFS	
**Surface processes**	Noah LSM		GFDL	
**Boundary conditions**	NCEP FNL Global analysis		NCEP GFS forecast	

**Table 2.** Vector track position errors (km).

**Time of model forecast**	**ARW**	**NMM**	**HWRF**
00_27 Aug 2005	32	21	16
06_27 Aug 2005	59	77	25
12_27 Aug 2005	71	120	56
18_27 Aug 2005	96	112	81
00_28 Aug 2005	132	152	99
06_28 Aug 2005	154	224	102
12_28 Aug 2005	158	278	96
18_28 Aug 2005	146	333	96
00_29 Aug 2005	132	380	70
06_29 Aug 2005	141	436	81
12_29 Aug 2005	194	458	60
18_29 Aug 2005	234	442	35
00_30 Aug 2005	262	490	16
